# Hedgehog Signaling Pathway and Autophagy in Cancer

**DOI:** 10.3390/ijms19082279

**Published:** 2018-08-03

**Authors:** Xian Zeng, Dianwen Ju

**Affiliations:** 1 Department of Microbiological and Biochemical Pharmacy & The Key Laboratory of Smart Drug Delivery MOE, School of Pharmacy, Fudan University, Shanghai 201203, China; zengxian@u.nus.edu; 2 Department of Pharmacy, Faculty of Science, National University of Singapore, Singapore 117543, Singapore

**Keywords:** hedgehog, autophagy, crosstalk, drug combination, drug resistance, precision medicine, cancer therapy

## Abstract

Hedgehog (Hh) pathway controls complex developmental processes in vertebrates. Abnormal activation of Hh pathway is responsible for tumorigenesis and maintenance of multiple cancers, and thus addressing this represents promising therapeutic opportunities. In recent years, two Hh inhibitors have been approved for basal cell carcinoma (BCC) treatment and show extraordinary clinical outcomes. Meanwhile, a series of novel agents are being developed for the treatment of several cancers, including lung cancer, leukemia, and pancreatic cancer. Unfortunately, Hh inhibition fails to show satisfactory benefits in these cancer types compared with the success stories in BCC, highlighting the need for better understanding of Hh signaling in cancer. Autophagy, a conserved biological process for cellular component elimination, plays critical roles in the initiation, progression, and drug resistance of cancer, and therefore, implied potential to be targeted. Recent evidence demonstrated that Hh signaling interplays with autophagy in multiple cancers. Importantly, modulating this crosstalk exhibited noteworthy capability to sensitize primary and drug-resistant cancer cells to Hh inhibitors, representing an emerging opportunity to reboot the efficacy of Hh inhibition in those insensitive tumors, and to tackle drug resistance challenges. This review will highlight recent advances of Hh pathway and autophagy in cancers, and focus on their crosstalk and the implied therapeutic opportunities.

## 1. Introduction

Hedgehog (Hh) signaling pathway regulates multiple key developmental processes, including embryogenesis and cell proliferation, cell fate determination, and tissue patterning in vertebrates [1,2]. In the adult, controlled Hh signaling activity is crucial for wound healing, tissue regeneration, and homeostasis maintenance [3,4]. However, ectopic Hh pathway activation has been documented to be responsible for tumorigenesis, progression, metastasis, and drug resistance of various cancers, including basal cell carcinoma (BCC), medulloblastoma (MB), and many other solid and hematological tumors [5]. Due to these extensive involvements in cancers, the Hh pathway has emerged as an attractive target for cancer therapy. Over the last two decades, mountains of efforts have been dedicated to the fundamental understanding of Hh pathway, as well as the development of clinical-level Hh pathway modulators. Thus far, two Hh pathway inhibitors, vismodegib and sonidegib, have been approved by the US Food and Drug Administration (FDA) for treatment of BCC in 2012 and 2015, respectively. In parallel, a series of novel small molecules are being developed under ongoing clinical trials or preclinical investigations [6].

However, remarkably, clinical benefits of targeting the Hh pathway are only limited to a few cancers, such as BCC and MB [7,8]. Results of clinical trials in other cancers indicated that Hh pathway inhibitors failed to show significant clinical benefit. For instance, a phase I study showed that 60% of BCC patients (19 out of 33) exhibited a partial or complete response to vismodegib treatment, and opposite to this encouraging outcome, none of 34 patients with other solid tumors showed partial or complete responses [9]. Moreover, clinical trials of the combination of vismodegib and gemcitabine demonstrated that the addition of vismodegib did not enhance overall response rate and extend patient survival time in metastatic pancreatic cancer, though the Hh signaling activity has been significantly decreased [10,11]. These unexcepted failures imply the complexity of Hh signaling responses and its varied roles in cancers. In addition, acquired resistance is emerging as a big challenge that compromises the clinical benefits of Hh inhibitors, and data suggested that mutations of Hh signaling components [12] and the activation of bypass signaling, such as phosphatidylinositol 3-kinase (PI3K) pathway [13], represent the potential mechanisms. Importantly, these findings indicated that combined inhibition of Hh signaling and other oncogenic pathways may be effective for improving the antitumor efficacy of Hh inhibitors and circumventing drug resistance.

Autophagy, a conservative metabolic process for degradation and recycling of damaged organelles or misfolded proteins, is responsible for maintaining cellular homeostasis and adapting to changes and stimuli imposed by the intracellular or surrounding environmental conditions [14]. Mounting evidence has demonstrated that autophagy plays critical roles in carcinogenesis, progression, and treatment resistance of various cancers, and has thus emerged as a possible anticancer target for alone or in conjunction with other targets [15,16]. Based on these advances, about 50 clinical trials have been proposed or are undergoing to evaluate the anticancer activity of combining autophagy modulators with chemotherapies or targeted drugs, according to the records on the ClinicalTrials.gov [17]. Recent studies have revealed that the Hh signaling pathway interplays with autophagy in various conditions. Some studies have attempted to elucidate the underlying molecular mechanisms. Moreover, co-manipulating Hh pathway and autophagy has been demonstrated to be able to show favorable anticancer effects in both in vitro and in vivo models. This review will briefly highlight the current clinical updates of Hh signaling and autophagy in cancer therapy, then focus on the crosstalk between Hh pathway and autophagy, and review the preclinical attempts that combined targeting Hh signaling and autophagy in the view of potential therapeutic implications for cancer therapy.

## 2. Hedgehog Signaling Pathway at a Glance

Attempts to decipher the transduction mechanisms of Hh pathway have never been stopped since the first disclosure of the Hh pathway in 1980 by Nüsslein-Volhard and Wieschaus [1]. Understanding of the biological fundamentals of Hh pathway has accelerated its translation to clinical therapeutics. Below sections will firstly summarize current understanding on the Hh signaling cascades, followed by a brief update of clinical advances in targeting Hh signaling, especially combined with other oncogenic pathways, for cancer therapy.

### 2.1. Canonical and Non-Canonical Hedgehog (Hh) Signaling Transductions

Canonical Hh signaling pathway involves multiple major components: the Hh ligand family member proteins (include three isoforms: Sonic hedgehog (SHH), Indian hedgehog (IHH) and Desert hedgehog (DHH)), the 12-transmembrane domain receptor protein Patched (PTCH) which locates in the primary cilium, the 7-transmembrane G-protein coupled receptor Smoothened (SMO), the suppressor of fused protein (SUFU) in cytoplasm, and the glioma-associated oncogene (GLI) transcription factors. In the absence of Hh ligands, PTCH represses the accumulation of SMO on the primary cilia, thus resulting in the inactivation of the downstream cascade. By contrast, the presence of Hh ligands will lead to the binding of Hh to PTCH so that PTCH unleashes SMO, which consequently triggers downstream activation of GLI transcription factors. GLI transcription factors consist of three proteins: GLI1, GLI2, and GLI3. Among them, GLI1 only has its activator form, while GLI2 and GLI3 have both activators (GLI2^A^ and GLI3^A^) and repressor forms (GLI2^R^ and GLI3^R^). GLI transcription factors are the nuclear executors at the end of the Hh pathway which are responsible for regulating downstream target genes.

Compared with canonical Hh signaling, the non-canonical Hh pathway, whose activation is independent of Hh ligands, is less well-studied. Current understanding of noncanonical Hh signaling suggests two distinct types: type I—SMO-independent but GLI-dependent, and type II—SMO-dependent but GLI-independent. Much evidence has demonstrated that GLI1 can be activated by other signaling pathways, such as PI3K/Protein kinase B (AKT)/mammalian target of rapamycin (mTOR) [18,19,20], transforming growth factor-β (TGFβ)/Sma- and Mad-related Protein 3 (SMAD3) [21,22], Raf serine/threonine kinase (RAF)/Meiosis-specific serine/threonine-protein kinase (MEK)/Mitogen-activated protein kinase (MAPK) [23,24], and Kirsten rat sarcoma 2 viral oncogene homolog (KRAS)/androgen receptor (AR) pathways [25], in a SMO-independent manner. These studies also indicated that such activation of type I noncanonical Hh activity is closely related to tumorigenesis, progression, and drug resistance in multiple cancers. Type II non-canonical Hh signaling activation has been observed to positively regulate Wingless-related integration site (WNT) pathway in colon cancer stem cells [26] and decrease intracellular levels of 3′,5′-cyclic adenosine monophosphate (cAMP) in airway epithelial cells [27]. Moreover, the coexistence of canonical and noncanonical Hh signaling has also been reported. For example, in multiple myeloma (MM) cells, SMO inhibitor sonidegib downregulated GLI1 and PTCH1 and led to significant cell death, indicating that the canonical Hh signaling exists and contributes to MM cell proliferation. Meanwhile, both cytosolic and nuclear GLI1 could be detected, and the nuclear localization of GLI1 could be completely abolished by a GLI1 inhibitor forskolin, suggesting the non-canonical activation of Hh signaling in MM cells [28].

### 2.2. Clinical Advances in the Combination of Hh Signaling Inhibition and Other Targeted Therapies

Good understanding of mechanisms and roles of interplay between Hh pathway and other oncogenic pathways opens new opportunities for rational design of combination cancer therapies. In recent years, there are at least 16 clinical trials that have been proposed to prove the combinatory concept, as shown in Table 1 (combination with chemotherapies are not included). The majority of them attempt to combine SMO inhibitors with other targeted drugs. These combined drugs include small molecular inhibitors of the Janus kinase (JAK) pathway, PI3K, mTOR, and epidermal growth factor receptor (EGFR), and macromolecular agents that target EGFR, vascular endothelial growth factor (VEGF), and programmed death-ligand 1 (PD-1), for treatment of various solid tumors. Besides, SMO inhibitors are combined with BCR-ABL modulators for chronic myeloid leukemia (CML) treatment. Only three proposals combine GLI inhibitors with others, which is easy to understand because there are no GLI targeted agents that have been approved for clinical use, so far. In fact, the arsenic trioxide (ATO) used in these combinations has been approved by US FDA for acute promyelocytic leukemia (APL) treatment. Recent studies revealed its potent inhibitory effect on GLI, and therefore, it has been suggested as a promising Hh signaling inhibitor [29,30]. In addition to ATO, there is another repositioning drug, itraconazole (a US FDA approved antifungal drug), that was recently found to exhibit potent Hh inhibitory effect by targeting SMO in multiple cancers [31,32]. More importantly, both ATO and itraconazole were reported to be able to overcome SMO mutation-derived drug resistance [33], making the clinical trial results of use alone, or in conjunction with other drugs, to be expected. Combined inhibition of Hh signaling and other oncogenic pathways has been suggested as a promising strategy to improve Hh inhibition effect and overcome drug resistance. The results of a phase I clinical trial that combined saridegib with cetuximab (NCT01255800) have shown satisfactory toxicity profile and signs of antitumor activity of the combination [34]. Further clinical evaluation of this combination, as well as results of other clinical trials listed in Table 1, will be very anticipated.

## 3. Autophagy in Cancer

### 3.1. Regulation of Autophagy in Cancer Cells

Macroautophagy (autophagy hereafter), a highly conserved intracellular process that degrades harmful or superfluous cellular materials to maintain homeostasis or respond to stimuli, involves three major steps: (1) formation of the autophagosomes, double-membraned vesicles which engulf proteins or organelles need to be degraded; (2) fusion of autophagosomes with lysosomes; and (3) degradation of engulfed materials by enzymes in lysosomes. To date, there are more than 30 autophagy-related genes (ATGs) have been found to orchestrate these processes, and the underlying molecular mechanisms have been comprehensively described in most recent reviews [14,35]. Briefly, in the initial autophagosome formation, the Unc-51-like kinase (ULK) complex (consists of ULK1, ULK2, ATG13, RB1-inducible coiled-coil protein 1 (RBCC1), and ATG101) is activated by upstream signals, which further activates class III PI3K complex (includes Beclin1, ATG14, VPS34, and p150). Besides, ATG9, ATG3, ATG7, and ATG12–ATG5–ATG16L1 complex also contribute to membrane elongation and autophagosome growth. Of note, in the autophagy formation process, the conversion of LC3B-I to LC3B-II is a hallmark of autophagosome formation and is widely used as a monitoring marker to track autophagy initiation [35]. It is well established that basal level autophagy is required for almost all mammalian cells, and a variety of stimuli or stress, such as hypoxia [36], nutrient shortage [37,38], and drug treatment [39,40], can upregulate autophagy levels in various types of normal or cancer cells.

### 3.2. Context-Dependent Roles of Autophagy in Cancer

A large volume of literature has demonstrated the role and mechanism of autophagy in tumorigenesis, progression, treatment, and drug resistance in cancers. It is now clear that autophagy plays varied roles in different scenarios. Evidence supports various lines of stories, such as that autophagy prevents/initiates cancer and autophagy leads to cell survival/cell death. Autophagy is required for normal antitumor immunosurveillance and is therefore useful for preventing cancer initiation before neoplasms are established [41,42]. Conversely, in established tumors, upregulated autophagy helps cancer cells to cope with intracellular stress and unfavorable environmental conditions resulting from rapid cell proliferation and tumor growth, representing a mechanism of cancer progression. Besides, countless evidence indicates that autophagy helps cancer cells to escape or resist a wide range of anticancer drugs. Based on these facts, a variety of clinical trials have been proposed to enhance anticancer efficacy and circumvent drug resistance of targeted drugs (as shown in Table 2) and chemotherapeutics by combining autophagy inhibitors. Detailed clinical updates of combining autophagy in cancer therapies have been discussed in recent reviews [16,43]. Of course, autophagy does not always act as a bad guy in cancer treatment. Autophagy plays critical roles in activating anticancer immune responses in radiotherapy [44] and immunogenic chemotherapy [45]. Taken together, current evidence revealed that roles of autophagy in cancers are context-dependent, and further research on these topics will be helpful to accelerate the clinical application of modulating autophagy in cancer therapy.

## 4. Crosstalk between Hedgehog Signaling Pathway and Autophagy

**Figure 1:**
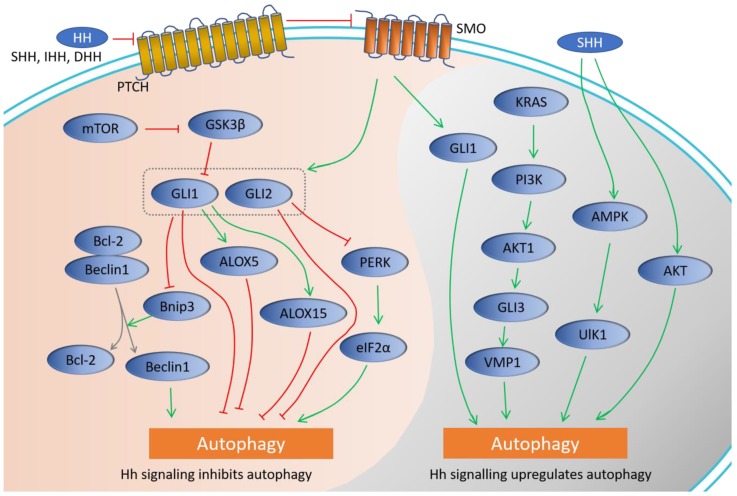
Crosstalk between Hh signaling and autophagy based on current evidence. Green arrow: upregulate; Red line: inhibit; Box with dash line: both GLI1 and GLI2 were involved in Bnip3 inhibition. Gray arrows: dissociation of Bcl-2/Beclin1 complex.

As one of the major players in the cell signaling system, Hh pathway interplays with multiple oncogenic pathways, such as PI3K/AKT/mTOR, RAF/MAPK/ERK, and KRAS pathways [46,47,48]. Although roles and mechanisms of autophagy or Hh signaling alone in human health and diseases have been intensively studies since decades ago, crosstalk between them has not been revealed until recent years. In the following sections, we will summarize current evidence of crosstalk between Hh pathway and autophagy by two categories (as depicted in Figure 1): Hh signaling inhibits autophagy and Hh signaling promotes autophagy.

### 4.1. Hh Signaling Inhibits Autophagy

The inhibitory effect of Hh signaling on autophagy has been found in normal and cancer cells from various tissue origins. Jimenez-Sanchez et al. systematically demonstrated that the inhibitory effect of Hh signaling on autophagy is governed by the GLI2–PERK–eIF2α axis. Inhibiting Hh signaling by overexpression of PTCH1 or PTCH2 increased autophagy level in both HeLa cells and mouse embryonic fibroblasts (MEFs). Similar results were also observed under the *SMO* knockdown condition. Accordingly, activating Hh signaling by overexpression of SMO or treatment with SMO agonist purmorphamine (all discussed Hh modulators have been summarized in Table 3) impaired autophagy. These data collectively confirmed that Hh signaling inhibits autophagy. Consistently, this inhibitory effect was also confirmed in *Drosophila*. Moreover, the authors further revealed that Hh signaling exerts its inhibitory effect by inhibiting the Protein kinase R (PRK)-like endoplasmic reticulum kinase (PERK) and reducing the phosphorylation of eukaryotic initiation factor 2α (eIF2α) [49]. Indeed, PERK–eIF2α has been implicated to be essential for autophagy induction under several conditions, such as ER stress and hypoxia [50,51]. Interestingly, purmorphamine impaired autophagy in *GLI1*^−/−^ or *GLI3*^−/−^ mice MEFs in the same way as in *GLI* wild-type counterparts, whereas it failed to inhibit autophagy in *GLI2*^−/−^ MEFs, indicating that such inhibitory effect of Hh signaling on autophagy depends on *GLI2*, but not *GLI1* or *GLI3*. Importantly, this evidence highlighted the central role of *GLI2* in Hh–autophagy crosstalk [49].

In several cancer cell lines: H4 (glioma), ES2 (ovarian cancer), MKN45 (gastric cancer), and HT29 (colon cancer cells), by indiscriminate analysis of more than 30,000 transcripts using microarray chips, many ATGs were found to be differentially upregulated after inhibiting Hh signaling with GANT61 treatment. Further experiments validated the activation of autophagy induced by Hh signaling inhibition in these four cell lines. Moreover, activation Hh signaling by N-SHH, a recombinant SHH peptide, subdued autophagy level and partially rescued cancer cells from Hh inhibition-induced cytotoxicity, suggesting the role of autophagic cell death [64].

It has also been reported that Hh signaling was active in hepatocellular carcinoma (HCC) cells, inhibition of Hh signaling by GLI1/2 inhibitor GANT61-induced autophagy, while activation of Hh signaling by Hh ligand protein or Hh agonist prevented the autophagy induction. Interestingly, inhibiting GANT61-induced autophagy could hamper GANT61-induced apoptosis, cytotoxicity, and in vivo tumor suppression effect, indicating the cell-killing role of GANT61-induced autophagy in HCC cells [65].

Abnormal activation of Hh signaling has been observed in breast cancer and implicated in drug resistance [66,67]. Itraconazole, a clinically used antifungal drug which is repurposed for cancer therapy, has been demonstrated to be able to kill cancer cells via inhibiting Hh signaling [31] and inducing apoptosis and cell cycle arrest [32]. A recent study showed that inhibition of Hh signaling by itraconazole induced cytotoxicity, apoptosis, tumor shrinkage, and autophagy in breast cancer in vitro cell lines or in vivo mouse models. Moreover, inhibiting autophagy by 3-MA or *ATG5* silencing attenuated itraconazole-induced cell death, suggesting that Hh inhibition-induced autophagy played a role in autophagic cell death [66]. In addition, another study demonstrated that itraconazole induced autophagy in glioblastoma cell lines U87 and C6, via repressing AKT1–mTOR signaling. Inhibiting autophagy by ATG5 or Beclin1 silencing rescued cells from itraconazole-induced cell death, indicating the cell-killing role of autophagy in this condition. However, the authors did not determine the change of Hh signaling activity during these processes [68].

Also, in lung cancer, the Hh signaling activation has been detected in several cell lines and patient samples, and therefore has been evaluated as a promising therapeutic target [69,70,71]. A recent study observed the Hh–autophagy relationship in A549 and 95D lung cancer cell lines. Bisdemethoxycurcumin (BDMC), a phytochemical that exhibits potent anticancer effect, reduced cell viability and induced apoptosis in A549 and 95D cells. To further probe the underlying mechanism, this study found that BDMC induced autophagy. Importantly, inhibiting Hh signaling by GLI1 siRNA or cyclopamine treatment significantly enhanced BDMC-induced autophagy, indicating that the induction of autophagy was partially inhibited by Hh signaling under BDMC treatment [72]. Most recently, results from our own lab described the interaction between Hh signaling and autophagy in lung adenocarcinoma. Our data indicated that SMO antagonist vismodegib failed to induce obvious cell death in A549 and NCI-H1975 cells and tumor shrinkage in xenograft mouse model but triggered marked autophagy [73]. Our further experiments showed that inhibiting vismodegib-induced autophagy by pharmacological inhibitors or gene knockdown could reboot the sensitivity of lung adenocarcinoma to vismodegib both in cell lines and in vivo models, suggesting autophagy might be a compensatory mechanism of Hh inhibition in lung cancer [74].

Consuelo Amantini et al. has reported that capsaicin, a plant-derived natural product which exerts anticancer potential, induced significant autophagy and upregulated expression of PTCH2 (a negative regulator of Hh signaling) in bladder cancer cell lines. This activation of autophagy resulted in epithelial–mesenchymal transition (EMT) and chemoresistance in bladder cancer cells. Importantly, silencing the *PTCH2* gene could weaken the autophagy induction, suggesting the involvement of Hh signaling in capsaicin-induced autophagy in bladder cancer cells [75].

In pancreatic cancer cells, inhibiting Hh signaling by GANT61-induced autophagy both in in vitro cells and in vivo mouse model, while activating Hh signaling by N-SHH transfection reduced autophagy. Moreover, the combination with 3-MA in vitro and in vivo showed an impaired antitumor activity of GANT61, suggesting that autophagy acted as a cell-killing role [76]. Hh signaling has also been demonstrated to suppress tumor growth by promoting the formation of fibroblast-rich stroma in pancreatic carcinoma [77]. Besides, a recent study delineated that the stromal stellate cells provoked pancreatic tumor growth by supplying alanine to overcome nutrient-poor tumor microenvironment, and the increased secretion of alanine was achieved by induction of autophagy [78]. Based on these two studies, it can be inferred that Hh signaling represses autophagy in stromal stellate cells to restrain tumor growth by subduing autophagy-induced alanine supplement. These studies also indicated that autophagy plays distinct roles in pancreatic stroma and pancreatic cancer cells.

In chondrosarcoma, the activation of the Hh signaling pathway has also been observed and has been suggested as a potential therapeutic target [79,80]. Expression of PTCH1, SMO, and GLI1 proteins was detected in chondrosarcoma patient tissues and three chondrosarcoma cell lines (HC-a, SW1353, and JJ012), but not in normal articular cartilage tissues. Interestingly, silencing of *GLI1* by siRNA significantly decreased cell viabilities and induced autophagy at the same time. Of note, data from this study indicated that activation of Hh signaling in chondrosarcoma cancer cells is type I noncanonical Hh signaling (GLI1-dependent and SMO-independent) since the Hh pathway activity can be diminished by *GLI1* knockdown, but not by cyclopamine treatment. The authors proposed the GLI1–mTOR–autophagy cascade by proving the requirement of mTOR dephosphorylation in this process. Further results depicted that co-treatment with 3-MA and CQ or silencing of *ATG5/Beclin1* could reverse GLI1 inhibition-induced cell death, indicating the cell-killing effect of autophagy in this case [81]. In agreement with these results, another study reported that arsenic trioxide (ATO), a GLI targeted Hh signaling inhibitor, induced autophagy in SW1353 cell line [82]. 

Rhabdomyosarcoma, the most common soft tissue sarcoma in young children, has been characterized by abnormal activation of Hh signaling [83,84]. A recent study has depicted that inhibition of Hh signaling using LDE225 or cyclopamine could activate autophagy in RUCH-2 and Rh41 rhabdomyosarcoma cell lines [85].

Neuroblastoma (NB) is the most common extracranial childhood solid tumor, accounting for ~15% of cancer-related childhood mortality [86]. Amplification of *MYCN* oncogene in NB is consistently correlated with high-risk NB stage, poor clinical outcome, and therapy-resistance [87,88]. Blocking Hh signaling showed antitumor efficacy both in in vitro and in vivo models, and therefore, has been suggested as a potential NB treatment strategy [89]. Whereas several studies have suggested that compared with *MYCN*-non-amplified NB cells, *MYCN*-amplified NB cells are relatively insensitive to Hh inhibition. To explore the underlying mechanism, Jing Wang et al. proposed autophagy activation induced by Hh inhibition as a mechanism of this compromised treatment effect. GANT61, a GLI1/2 inhibitor, induced significant cytotoxicity in *MYCN*-non-amplified NB cells without induction of autophagy. Conversely, GANT61 failed to induce cytotoxicity in *MYCN*-amplified NB cells, but triggered obvious autophagy. Inhibition of autophagy by 3-MA or *ATG5/ATG7* silencing could enhance GANT61-induced cell death, suggesting that the cytoprotective effect of autophagy played a critical role in this case. Collectively, this suggested that autophagy may be responsible for the insensitivity of *MYCN*-amplified NB cells to Hh inhibition [90]. Most recently, data from the same research lab indicated that PERK is a key mediator of GANT61-induced autophagy in *MYCN*-amplified NB cells [91], which is in line with results from MEF models [49].

The development of medulloblastoma (MB), another prevalent brain tumor in young children, can be generally attributed to four types of dysregulations: WNT/β-catenin, SHH, MYCN, and heterogeneous genes [92]. Amplification of Hh signaling has been well characterized, and several clinical trials have been initiated to evaluate the efficacy of the SMO antagonist vismodegib on MB patients (Table 3). Moreover, the PI3K/mTOR pathway has been demonstrated to play crucial roles in SHH- and MYCN-driven MB [13,93]. Importantly, Hh signaling can be activated by PI3K/mTOR in several cancers [18,19]. A most recent study reported that the conjunction of Hh inhibitor and PI3K/mTOR inhibitor exhibited significantly enhanced antitumor effects both in cell lines and xenograft mouse model of Hh-driven and MYCN-driven MB, underpinning the importance of Hh and PI3K/mTOR in MB [94]. In addition, it is well documented that the induction of PI3K/mTOR pathway halts autophagy, while inhibition of the PI3K/mTOR pathway stimulates autophagy [35]. Taken together, autophagy might be involved in the crosstalk between Hh and PI3K/mTOR in MB.

Resistance to ABL tyrosine kinase inhibitors is a major obstacle for improving clinical outcomes of chronic myeloid leukemia (CML) treatment. Both deregulations of Hh signaling and autophagy have been demonstrated to be involved in CML drug resistance [95,96,97,98]. Our study indicated that SMO antagonist vismodegib induced autophagy in several BCR-ABL^+^ CML cell lines, including K562 and BaF3 cell lines harboring the T315I mutation of *BCR-ABL* fusion gene (*BCR-ABL*) (an infamous drug resistance mutation in CML). Knockdown of SMO also led to an obvious elevation of autophagy level. Data from this study also suggested that inhibition of AKT/mTOR signaling pathway was involved in the autophagy induction. Most importantly, co-inhibiting Hh signaling and autophagy by the combination of vismodegib and CQ could induce robust apoptosis in BaF3-BCR-ABL^T315I^ cells which are resistant to CML drugs, such as imatinib and dasatinib, highlighting a possible way to overcome drug resistance in BCR-ABL^+^ CML patients [99]. Apart from CML cells, our recent study also suggested that in B-cell non-Hodgkin’s lymphoma (B-NHL) cell line Raji, inhibition of Hh signaling by vismodegib also triggered significant autophagy and blocking autophagy could sensitize B-NHL cells to vismodegib [100]. Moreover, a study from another group observed that inhibition of Hh signaling by LDE225 evoked autophagy in mantle cell lymphoma. Data from this study supported that upregulation of CXCR4 expression was involved in the autophagy induction, as *CXCR4* knockout cells failed to initiate autophagy upon LDE225 treatment. At the same time, the combination with 3-MA significantly enhanced LDE225-induced cell cytotoxicity, revealing the cytoprotective role of autophagy in this case [101]. These data collectively suggested that in addition to solid tumors, modulating Hh–autophagy interplay may be also useful for improving the anticancer effect and overcoming drug resistance in hematologic malignancies.

In addition to cancer cells, Hh-regulated autophagy has also been reported in other human cell types. Junpeng Huang et al. reported that exposure to hexavalent chromium (Cr(VI)), a class of compounds which are recognized as human carcinogens and related to lung cancer risk, upregulated *GLI2* gene expression, and such upregulation prevented the induction of autophagy in human bronchial epithelial cells. Inhibition of GLI2 by GANT61 or *GLI2* siRNA transfection reversed the autophagy inhibition. These results underscored the impact of GLI2 on autophagy inhibition [102]. Besides, activation of GLI2 has been reported to be involved in fibrogenesis, a process involves the transdifferentiation of human primary fibroblasts into myofibroblasts. In this process, the reduced autophagy level co-occurred with GLI2 activation, accordingly, inhibition of GLI2 with GANT61 counteracted the autophagy inhibition in human primary fibroblasts. These data supported the idea that GLI2 inhibits autophagy. This study also proposed that eIF2α may link Hh signaling and autophagy, since the inhibition of eIF2α phosphorylation was correlated with GLI2 activation [103]. Hepatic stellate cells (HSCs) are critical for fibrogenic progression in non-alcoholic steatohepatitis. Recent studies have reported a distinct relationship between Hh signaling and autophagy in HSCs. Xuyou Liu et al. reported that inhibition of Hh signaling induced autophagy in HSCs. Inhibition of Hh signaling by cyclopamine or *PTCH1*/*GLI1* siRNA led to increased autophagy level, and stimulation of Hh signaling by purmorphamine reduced autophagy, confirming the negative regulation of Hh signaling on autophagy in HSCs [104]. In supporting this observation, another study has reported that, in hepatic stellate cell line LX-2, inhibition of Hh signaling by GANT61 induced autophagy, which plays a cytoprotective role and relies on the induction of endoplasmic reticulum stress [105]. In contrast to these two studies, Nana Duan et al. found that the palmitic acid exposure led to activation of both Hh signaling and autophagy in human immortalized HSC, rat BSC-C10, and primary rat HSC cells. Accordingly, inhibition of Hh signaling by LDE225 suppressed autophagy induction [106]. These controversial results may be attributed to varied study conditions, such as different cell origins and perturbation agents.

Furthermore, it has been suggested that the crosstalk between Hh signaling and autophagy also plays roles in pathogen–host interactions in infective diseases. Autophagy plays a crucial role in both innate and adaptive immune systems [41,107]. Host cells can elevate autophagy to restrict intracellular pathogens and promote the major histocompatibility complex (MHC) class II presentation of the pathogen-derived antigens, servicing as a major mechanism of infection prevention [108,109,110]. However, pathogens can evade this protection mechanism by inhibiting autophagy level in host cells. Sahana Holla et al. reported that some bacteria species, such as *Mycobacteria*, *Shigella*, and *Listeria*, could selectively inhibit autophagy in macrophages by provoking robust Hh signaling activation. Their data demonstrated that Hh signaling pathway repressed autophagy via the upregulation of arachidonate 5-lipoxygenase (*ALOX5*) or arachidonate 15-lipoxygenase (*ALOX15*) gene expression, which negatively regulates autophagy. The authors proposed the mTOR–GSK3β–GLI1–ALOX5/ALOX15–autophagy axis in this regulation [111].

### 4.2. Hh Signaling Upregulates Autophagy

Many studies have observed the activating effect of Hh signaling on autophagy. For example, Luis Milla et al. reported that Hh antagonist cyclopamine prevented autophagy activation in neuroblastoma cell line SHSY5Y. Moreover, in the SHH-sensitive cell line C3H10T1/2, deprivation of SHH ligand protein in culture medium abolished Hh signaling activation and, at the same time, led to decreased protein expression of ATG5, a key protein for autophagy activation. These findings suggested that Hh signaling mediated the activation of autophagy in neuroblastoma cell lines [112]. Oncogene *KRAS* could induce autophagy to promote tumorigenesis and cancer progression and the KRAS–PI3K–AKT1–GLI3–VMP1 axis has been postulated as an underlying mechanism [113]. In this case, GLI3 was able to activate autophagy via upregulating expression of the VMP1, a protein essential for autophagy activation, whereas, GLI3-regulated autophagy was SMO-independent.

Tumor-initiating cells (TICs), also known as cancer stem cells (CSCs), are a small subpopulation of cells in the tumor which can be recognized by several cell surface markers, such as CD133, CD24, CD44, and Bmi-1 [114]. TICs exert the capacity of self-renewal, differentiation, and tumor initiation, and play critical roles in tumorigenesis, tumor growth and maintenance, radio- and chemotherapy resistance, and tumor relapse [115,116,117]. Importantly, Hh signaling regulates TICs in many cancers, including breast cancer, pancreatic cancer, gastric cancer, and brain cancers [118,119,120,121,122], representing a promising therapeutic target by controlling TICs. For example, it has been reported that activated Hh signaling suppressed Fas and DR4/5 expression, stimulated Bcl-2 and PDGFR expression in pancreatic TICs, and thus promoted cell growth, while inhibition of Hh led to apoptosis [118]. Activation of Hh signaling promoted TIC growth via modulating Bmi-1 gene expression in human breast cancer [119]. Moreover, it has been revealed that autophagy plays critical roles in TIC maintenance and growth. For instance, in breast cancer, activation of autophagy suppressed tumor development in a TIC-poor enriched tumor model but played a promotional role in TIC maintenance [123], revealing the unique role of autophagy in breast cancer TICs for growth and survival. In pancreatic cancer, Haitao et al. reported that under hypoxia, the upregulated HIF-1α induced autophagy which mediated the conversion of non-stem pancreatic cancer cells to stem-like cells to maintain the equilibrium of TICs [124]. In addition, another study revealed that hypoxia activated HIF-1α expression, which subsequently led to upregulated of Hh signaling, and finally made pancreatic cancer more aggressive and resistant to treatment [125]. Taken together, it might be speculated that Hh signaling promotes autophagy in pancreatic TICs, and both of them collectively contribute to growth and survival of pancreatic cancer TICs.

Strictly controlled Hh signaling activity is essential for development, repair, and homeostasis of many tissues, including cardiovascular tissue, intestinal epithelium, and neurons [2]. In mouse vascular smooth muscle cells, a research group reported that adding SHH ligands or overexpressing SHH gene instigated autophagy. Activated Hh signaling promoted cell proliferation, and this effect partially depended on the activation of autophagy [126]. By establishing an SHH intestinal epithelial conditional knockout mice model, Jessica G.S. et al. indicated that Hh signaling plays a critical role in homeostasis of intestinal ileum, and loss of SHH signaling lead to the decrease of autophagy in intestinal ileum [127]. Qing Xiao et al. reported that Hh signaling activation by SHH agonist SAG protected cardiomyocytes H9C2 cells from injury under oxygen-glucose deprivation (OGD) condition. This protective role of SHH signaling was achieved by upregulation of autophagy through activating AMPK/UlK1 signaling [128]. Ronald S.P. and colleagues revealed that in hippocampal neurons, exposure to SHH ligand proteins led to significant upregulation of autophagy. Their further results demonstrated that the Class III PI3K was required for this autophagy activation since 3-MA could effectively prevent SHH ligand-induced autophagy [129]. However, the further molecular mechanism under this crosstalk in these cell types remains unclear.

It is worth noting that, in a patient-based analysis, key components of Hh signaling and autophagy have been suggested as significant prognostic biomarkers. In a study of 108 gastric patients, immunohistochemical staining indicated that both Beclin1 and GLI2 are highly expressed in adjacent normal gastric mucosa samples (86.5% and 100%, respectively) and adenocarcinoma samples (60.2% and 72.2%, respectively) [130]. Higher expression of GLI2 was correlated with lower primary tumor stage (T1-2 vs. T3-4, *p* = 0.036), no lymphatic invasion (absent vs. present, *p* = 0.034), and no tumor recurrence (absent vs. present, *p* = 0.011). Similarly, increased expression of Beclin1 was correlated with favorable prognostic variables. Moreover, the expression of GLI2 was highly correlated with Beclin1 expression level in patient samples. Hence, this study postulated that autophagy may be related to Hh signaling in gastric cancer, and together, Beclin1 and GLI2 expression level is a possible prognostic biomarker. However, the interaction between HH signaling and autophagy has not been investigated in this study. Importantly, this study provided unexcepted evidence that higher GLI2 expression could be a favorable prognostic marker, and Beclin1 acted as a tumor suppressor in gastric cancer. This is opposed to our conventional impression that activation of Hh signaling is related to cancer initiation, progression, and metastasis, indicating the complex roles of Hh signaling and autophagy in cancers again.

## 5. Combined Targeting Hh Pathway and Autophagy: A Therapeutic Opportunity for Cancer Therapy

As discussed in preceding sections, current studies indicated that how Hh signaling affects autophagy depends on specific research conditions, and Hh signaling exerts negative regulation on autophagy in the majority of these studies. As shown in Table 4, in some cases, such as CML [99] and lung cancer [73,74], Hh inhibition could induce cytoprotective autophagy, indicating the benefits of the combination of Hh pathway inhibitors with autophagy inhibitors. In such cases, inducing autophagy seems to be a bypass or compensatory mechanism when Hh signaling is inhibited, whereas other studies, such as HCC [65] and cases of chondrosarcoma [81], implicated the cell-killing role of Hh-related autophagy, suggesting that autophagy activators may be helpful. Collectively, these results presented the complex roles of Hh-related autophagy in cancers and whether inhibiting or activating, Hh-related autophagy is not a “one-size-fits-all” paradigm. In fact, the context-dependent roles of autophagy activation in cancer therapy have attracted intense attention and raised hot debates. Both inhibiting and activating autophagy, therefore, have been proposed to improve the anticancer activity of many drugs. Fortunately, in the view of the forthcoming precision/personalized medicine, the context-dependent roles of autophagy will not be an obstacle for manipulating autophagy in cancer therapy, because patients will be well stratified into subpopulations based on some indicative biomarkers which correspond to different optimal treatment strategies [131]. In such a desirable scenario in the future, autophagy may become one of these indicative biomarkers.

To this end, much attention has been paid to evaluate the potential applications of autophagy proteins, including Beclin1, LC3B, ATG7, MAPK8IP1, and SH3GLB1, as prognostic biomarkers in several cancer types, such as breast cancer, colon cancer, melanoma, and glioma [132,133,134,135,136,137,138]. For example, Park et al. analyzed the correlation of Beclin1 expression and overall survival of 178 colon cancer patients who were receiving 5-fluorouracil therapy, and their results demonstrated that Beclin1 expression (hazard ratio was 1.82) could be a prognostic biomarker to guide optimal patient stratification [134]. LC3B has also been suggested as a prognostic biomarker for both relapse-free survival and OS in breast cancer based on an immunochemistry analysis of 229 breast cancer patient specimens [135]. These studies collectively indicated the exciting prospect of integrating autophagy as a biomarker in clinical applications. Nevertheless, the detection of almost all these biomarkers will heavily rely on tumor specimen or biopsy, which limits their practicability in clinical usage given that the autophagy in cancer progression and treatment is a dynamic process and thus needs dynamic monitoring. Therefore, the development of new biomarkers which can be monitored in blood or other bodily fluids using noninvasive detection methods is extremely significant, and will largely improve the applicability of monitoring autophagy in clinical scenarios.

Although the role of Hh-related autophagy seems to be confused currently, encouraging results from *MYCN*-amplified neuroblastoma and BCR-ABL mutation CML studies underscored the possibility of co-modulating Hh signaling and autophagy to overcome drug resistance in different cancer domains. Acquired drug resistance is emerging as the main challenge for achieving optimal clinical outcomes of SMO-targeted drugs. Currently proposed concepts mainly focus on developing drugs with new chemical properties, designing combination strategies that co-target multiple components of Hh signaling, and combined targeting of Hh signaling and other oncogenic pathways. Given the wide occurrence of Hh–autophagy crosstalk and its critical roles in cancer cells, we suggest that taking Hh–autophagy crosstalk into consideration may represent an alternative way.

However, only a few studies have attempted to delineate the underlying molecular mechanisms of Hh–autophagy association. Lack of mechanistic understanding of crosstalk between Hh signaling and autophagy limits the therapeutic applications. Hopefully, with the advances in the understanding of how Hh–autophagy crosstalk can be utilized towards an improve anticancer effect, the concept of combined targeting Hh signaling and autophagy will be validated in more preclinical or clinical studies, which will ultimately benefit cancer therapy.

## 6. Conclusions

Much evidence indicates that cancer progression and relapse may not be abolished by inhibiting the Hh signaling pathway alone, and redundant bypass and crosstalk with other pathways should be taken into consideration in future therapeutics development. Hh signaling regulates autophagy in various cell or animal models, and such crosstalk exhibits distinct pathological or pharmacological roles. In some cases, autophagy likely acts as a bypass or compensatory mechanism, which will be activated to support cancer cell growth when Hh is blocked. In these scenarios, co-targeting Hh signaling and autophagy represents a promising option to improve clinical outcomes of Hh-targeted agents and circumvent drug resistance. Some of these co-targeting attempts have shown encouraging results in in vitro or in vivo models. However, induction of autophagy could also be useful for Hh-targeted therapy in other cases, since activation of autophagy could lead to autophagic cell death, indicating that stimulating autophagy may result in favorable outcomes. Before we can harness the Hh–autophagy crosstalk to design improved anticancer strategies, considerable research efforts are needed to gain a deeper understanding of the underlying molecular mechanisms.

## Figures and Tables

**Table 1:** Clinical trials of combination of Hedgehog (Hh) inhibitors and other targeted therapeutics. (Data from ClinicalTrials.gov [17]).

Combined Drugs	Combined Targets	Disease Indications	Clinical Trial Stage	Clinical Trial Status	ClinicalTrials.gov Accession
Sonidegib + Ruxolitinib	SMO + JAK1/2	Myelofibrosis	Phase 1/2	Active, not recruiting	NCT01787552
Sonidegib + Buparlisib	SMO + PI3K	Advanced Solid Tumors	Phase 1	Completed	NCT01576666
Sonidegib + Ribociclib	SMO + CDK4/6	Refractory or Recurrent Medulloblastoma	Phase 1	Recruiting	NCT03434262
Vismodegib + RO4929097	SMO + Gamma-Secretase	Advanced or Metastatic Sarcoma	Phase 1/2	Completed	NCT01154452
Vismodegib + Bevacizumab	SMO + VEGF	Metastatic Colorectal Cancer	Phase 2	Completed	NCT00636610
Vismodegib + Sirolimus	SMO + mTOR	Metastatic Pancreatic Cancer	Phase 1	Suspended	NCT01537107
Vismodegib + Pembrolizumab	SMO + PD-1	Metastatic or Unresectable Basal Cell Skin Cancer	Phase 1/2	Active, not recruiting	NCT02690948
Vismodegib + Erlotinib + Gemcitabine	SMO + EGFR	Metastatic Pancreatic Cancer	Phase 1	Active, not recruiting	NCT00878163
Saridegib + Cetuximab	SMO + EGFR	Recurrent Head and Neck Cancer	Phase 1	Completed	NCT01255800
Sonidegib + Nilotinib	SMO + BCR-ABL	Chronic or Accelerated Phase Myeloid Leukemia	Phase 1	Completed	NCT01456676
BMS-833923 + Dasatinib	SMO + BCR-ABL/SRC	Chronic Myeloid Leukemia	Phase 1/2	Completed	NCT01218477
BMS-833923 + Dasatinib	SMO + BCR-ABL/SRC	Chronic Myeloid Leukemia	Phase 2	Terminated	NCT01357655
ATO + Icotinib	GLI + EGFR	EGFR-TKI Resistant Non-Small Cell Lung Cancer	Phase 1	Unknown	NCT02066870
ATO + Gleevec	GLI + BCR-ABL	CML Who Fail Gleevec	Phase 2	Completed	NCT00250042
ATO + GO	GLI + CD33	Advanced Myelodysplastic Syndromes	Phase 2	Completed	NCT00274781
ATO + GO + ATRA	GLI + CD33	Acute Promyelocytic Leukemia	Phase 2	Recruiting	NCT01409161

Abbreviations: SMO: smoothened; JAK: Janus kinase; PI3K: phosphatidylinositol 3-kinase; CDK: cyclin-dependent kinase; VEGF: vascular endothelial growth factor; mTOR: mammalian target of rapamycin; PD-1: programmed death-ligand 1; EGFR: epidermal growth factor receptor; BCR-ABL: BCR-ABL fusion gene; SRC: Src kinase; GLI: glioma-associated oncogene; SRC: Src kinase family; GO: gemtuzumab ozogamicin; ATRA: all-trans retinoic acid; ATO: arsenic trioxide.

**Table 2:** Clinical trials of combination of autophagy inhibitors and other targeted therapeutics. (Data from ClinicalTrials.gov [17]).

Combined Drugs	Combined Targets	Disease Indications	Clinical Trial Stage	Clinical Trial Status	ClinicalTrials.gov Accession
Vorinostat + HCQ	HDAC + Autophagy	Advanced Solid Tumors	Phase 1	Recruiting	NCT01023737
Vorinostat + HCQ	HDAC + Autophagy	Advanced Cancer	Phase 1	Active, not recruiting	NCT01266057
Vorinostat + HCQ	HDAC + Autophagy	Colorectal Cancer	Phase 2	Recruiting	NCT02316340
Sorafenib + HCQ	VEGFR/PDGFR/RAF + Autophagy	Refractory or Relapsed Solid Tumors	Phase 1	Completed	NCT01634893
Sorafenib + HCQ	VEGFR/PDGFR/RAF + Autophagy	Hepatocellular Cancer	Phase 2	Recruiting	NCT03037437
RAD001 + HCQ	MTOR + Autophagy	Renal Cell Carcinoma	Phase 1/2	Active, not recruiting	NCT01510119
MK2206 + HCQ	AKT + Autophagy	Advanced Solid Tumors, Melanoma, Prostate or Kidney Cancer	Phase 1	Active, not recruiting	NCT01480154
Trametinib + HCQ	MEK1/2 + Autophagy	Advanced BRAF Mutant Melanoma	Phase 1/2	Unknown	NCT02257424
FOLFOX6/XELOX + Bevacizumab + HCQ	VEGF + Autophagy	Metastatic Colorectal Cancer	Phase 2	Completed	NCT01006369
Abiraterone + Navitoclax + HCQ	BCL-2/BCL-xL/BCL-w + Autophagy	Progressive Metastatic Castrate Refractory Prostate Cancer	Phase 2	Terminated	NCT01828476

Abbreviations: HCQ: hydroxychloroquine; HDAC: histone deacetylases; VEGFR: vascular endothelial growth factor receptor; PDGFR: platelet-derived growth factor receptor; FOLFOX: a chemotherapy regimen that consists of folinic acid, fluorouracil, and oxaliplatin; XELOX: a chemotherapy combination that combines capecitabine and oxaliplatin; BCL: B-cell lymphoma.

**Table 3:** Hh pathway modulators discussed in the current context.

Chemical Modulator Name	Target (Mode of Action)	Clinical Indication Examples	Maximum Developmental Stage
Vismodegib (GDC-0449)	SMO (Antagonist)	**Approved:** BCC **Clinical Trials:** Pancreatic ductal adenocarcinoma (NCT01096732); MB (NCT00939484, NCT01239316, NCT00822458); Advanced/metastatic sarcoma (NCT01154452); Ovarian cancer (NCT00739661, NCT00959647); AML (NCT01880437);	Approved
Sonidegib (Erismodegib, NVP-LDE225, LDE-225)	SMO (Antagonist)	**Approved**: BCC **Clinical Trials**: Prostate Cancer (NCT02111187); Pancreatic Adenocarcinoma (NCT01431794); Multiple Myeloma (NCT02254551, NCT02086552); Ovarian Cancer (NCT02195973); Breast Cancer (NCT01757327); Small Cell Lung Cancer (NCT01579929);	Approved
Saridegib (IPI-926, Patidegib)	SMO (Antagonist)	**Clinical Trials:** Solid Tumors (NCT00761696); Myelofibrosis (NCT01371617); Chondrosarcoma (NCT01310816); Metastatic Pancreatic Cancer (NCT01130142); Gorlin Syndrome (NCT02762084);	Phase 2
Arsenic Trioxide (ATO)	GLI (Antagonist)	**Approved:** Acute promyelocytic leukemia (APL) **Clinical Trials:** Non-Small Cell Lung Cancer (NCT00075426); CML (NCT00250042); AML (NCT00005795); Myelodysplastic Syndrome (NCT00225992);	Approved ^1^
Itraconazole	SMO (Antagonist)	**Clinical Trials:** BCC (NCT01108094);	Approved ^2^
Cyclopamine	SMO (Antagonist)	**Indication Evidence From In Vivo Studies:** Small Cell Lung Cancer [52]; Glioblastoma [53]; CML [54]; Medulloblastoma [55]; Prostate [56]; Digestive tract tumors [57]; Pancreatic cancer [58];	Experimental Stage
GANT61	GLI (Antagonist)	**Indication Evidence From In Vivo Studies:** Pancreatic cancer [59]; Breast cancer [60]; Prostate Cancer [61]	Experimental Stage
Glabrescione B (GlaB)	GLI1 ^3^ (Antagonist)	**Indication Evidence From In Vivo Studies:** MB [62]	Experimental Stage
SAG	SMO (Agonist)	**Indication Evidence From In Vivo Studies:** Pancreatic cancer [63] ^4^	Experimental Stage
Purmorphamine	SMO (Agonist)	--	--

^1^ ATO has been approved by US FDA for APL but was not based on a mechanism of Hh inhibition; ^2^ Itraconazole has been approved by US FDA for antifungal purpose, while recent studies revealed its Hh inhibitory effect; ^3^ GlaB achieves inhibitory effect on GLI1 activity through binding to GLI1 zinc finger so that impairs its interaction with DNA; ^4^ Activation of Hh signaling by SAG in pancreatic stroma to restrain tumorigenesis and progression.

**Table 4:** A brief summary of studies that combined targeting Hh pathway and autophagy.

Cell Lines Used (Related Diseases)	Role of Hh-Related Autophagy and Supporting Evidence	Therapeutic Implications	References
H4 (Glioma), ES2 (Ovarian cancer), MKN45 (Gastric cancer), HT29 (Colon cancer)	**Role**: Autophagic cell death; **Evidence:** Inhibiting autophagy by 3-MA or knockout *ATG5* partially rescued HH inhibition-induced cell proliferation.	Hh inhibitor + autophagy inducer	[64]
MCF-7, SKBR-3 (Breast cancer)	**Role:** Autophagic cell death **Evidence:** Inhibition of autophagy by 3-MA or *ATG5* silencing impedes itraconazole-induced cell death.	Hh inhibitors + autophagy inducer	[66]
A549, NCI-H1975 (Lung cancer)	**Role:** Cytoprotective; **Evidence:** Inhibiting autophagy by inhibitors CQ or *ATG5* or *ATG7* siRNA strengthened vismodegib-induced cytotoxicity in cell lines. Co-administration of vismodegib-induced tumor-shrinkage in xenograft mouse model.	Hh inhibitor + autophagy inhibitor	[73,74]
HC-a, SW1353, JJ012 (Chondrosarcoma)	**Role:** Autophagic cell death; **Evidence:** Inhibiting autophagy by inhibitors (3-MA or CQ) or gene knockdown (*ATG7* or *Beclin1* siRNA) prevented GLI1 inhibition-induced cell death.	Hh inhibitor + autophagy inducer	[81]
Huh7, Hep3B, HepG2 (Liver cancer)	**Role:** Autophagic cell death; **Evidence:** inhibition autophagy by 3-MA or *Beclin1* siRNA hampered GANT61-induced apoptosis and cytotoxicity; 3-MA co-administration weakened the tumor-shrinkage induced by GANT61 in Huh7 xenograft mouse model.	Hh inhibitor + autophagy inducer	[65]
MYCN-amplified NBL-W-S and SK-N-BE cell lines (neuroblastoma)	**Role:** Cytoprotective; **Evidence:** Inhibiting autophagy by 3-MA or gene knockdown (*ATG5* or *ATG7* siRNA) enhanced GANT61-induced apoptosis.	Hh inhibitor + autophagy inhibitor	[90]
K562, BaF3-BCR-ABL^WT^, BaF3-BCR-ABL^Y253F^, BaF3-BCR-ABL^T315I^ (drug resistant CML)	**Role:** Cytoprotective; **Evidence:** Inhibiting autophagy by CQ or gene knockdown (*ATG5* or *ATG7* siRNA) enhanced vismodegib-induced apoptosis.	Hh inhibitor + autophagy inhibitor	[99]
Raji (non-Hodgkin’s lymphoma)	**Role:** Cytoprotective; **Evidence:** Inhibiting autophagy by inhibitors (CQ or bafilomycin A) or *ATG5* siRNA enhanced vismodegib-induced apoptosis.	Hh inhibitor + autophagy inhibitor	[100]
SP53 (Mantle cell lymphoma), Jeko, REC1, Pt1, Pt2	**Role:** Cytoprotective; **Evidence:** Combination with 3-MA significantly increased LDE255-induced cytotoxicity.	Hh inhibitor + autophagy inhibitor	[101]
Hepatic stellate cell line LX-2 (Liver fibrosis)	**Role:** Cytoprotective **Evidence:** inhibiting autophagy with 3-MA or CQ can enhance GANT61-induced cytotoxicity.	Hh inhibitors + autophagy inhibitor	[106]
CFPAC-1 (pancreatic cancer)	**Role:** Autophagic cell death; **Evidence:** Inhibiting autophagy by 3-MA reversed GANT61-induced cytotoxicity in cell lines and anticancer effect in in vivo mouse model.	Hh inhibitor + autophagy inducer	[76]

## Abbreviations

3-MA	3-Methyladenine
AKT	Protein kinase B
AR	Androgen receptor
ATG	Autophagy-related protein
BCC	Basal cell carcinoma
BCL	B-cell lymphoma
BCR-ABL	BCR-ABL fusion gene
BDMC	Bisdemethoxycurcumin
cAMP	3′,5′-cyclic adenosine monophosphate
CDK	Cyclin-dependent kinase
CML	Chronic myeloid leukemia
CQ	Chloroquine
DHH	Desert hedgehog
EGFR	Epidermal growth factor receptor
ER	Endoplasmic reticulum
ERK	Extracellular signal-regulated kinase
FOLFOX	A chemotherapy regimen that consists of folinic acid, fluorouracil, and oxaliplatin
GLI	Glioma-associated oncogene
GSK	Glycogen synthase kinase
HCC	Hepatocellular carcinoma
HCQ	Hydroxychloroquine
HDAC	Histone deacetylases
Hh	Hedgehog
IHH	Indian hedgehog
JAK	Janus kinase
KRAS	Kirsten rat sarcoma 2 viral oncogene homolog
LC3	Microtubule-associated proteins 1A/1B light chain 3B
MAPK	Mitogen-activated protein kinase
MB	Medulloblastoma
MEF	Mouse embryonic fibroblasts
MEK	Meiosis-specific serine/threonine-protein kinase
mTOR	Mammalian target of rapamycin
OGD	Oxygen-glucose deprivation
PD-1	Programmed death-ligand 1
PDGFR	Platelet-derived growth factor receptor
PERK	PKR-like endoplasmic reticulum kinase
PI3K	Phosphatidylinositol 3-kinase
PTCH	Patched
RAF	Raf serine/threonine kinase
RBCC1	RB1-inducible coiled-coil protein 1
SHH	Sonic hedgehog
SMO	Smoothened
SUFU	Suppressor of fused
SMAD3	Sma- and Mad-related Protein 3
SRC	Src kinase
SUFU	Suppressor of fused
TGFβ	Transforming growth factor-β
ULK	Unc-51-like kinase
VEGF	Vascular endothelial growth factor
VEGFR	Vascular endothelial growth factor receptor
WNT	Wingless-related integration site
XELOX	A chemotherapy combination that combines capecitabine and oxaliplatin
